# Metabolomic Profiling Revealed Diversion of Cytidinediphosphate-Diacylglycerol and Glycerol Pathway towards Denovo Triacylglycerol Synthesis in *Rhodosporidium toruloides*

**DOI:** 10.3390/jof7110967

**Published:** 2021-11-13

**Authors:** Farha Deeba, Kukkala Kiran Kumar, Girish H. Rajacharya, Naseem A. Gaur

**Affiliations:** International Centre for Genetic Engineering and Biotechnology (ICGEB), New Delhi 110067, India; kiranbiotech92@gmail.com (K.K.K.); hr.girish014@gmail.com (G.H.R.)

**Keywords:** *Rhodosporidium toruloides*, triacylglycerol, metabolomics, biofuel

## Abstract

Oleaginous yeast *Rhodosporidium toruloides* has great biotechnological potential and scientific interest, yet the molecular rationale of its cellular behavior to carbon and nitrogen ratios with concurrent lipid agglomeration remains elusive. Here, metabolomics adaptations of the *R. toruloides* in response to varying glucose and nitrogen concentrations have been investigated. In preliminary screening we found that 5% glucose (*w*/*v*) was optimal for further analysis in *Rhodosporidium toruloides* 3641. Hereafter, the effect of complementation to increase lipid agglomeration was evaluated with different nitrogen sources and their concentration. The results obtained illustrated that the biomass (13 g/L) and lipid (9.1 g/L) production were maximum on 5% (*w*/*v*) glucose and 0.12% (NH_4_)_2_SO_4_. Furthermore, to shed lights on lipid accumulation induced by nitrogen-limitation, we performed metabolomic analysis of the oleaginous yeast *R. toruloides* 3641. Significant changes were observed in metabolite concentrations by qualitative metabolomics through gas chromatography-mass spectrometry (GC-MS) and liquid chromatography-mass spectrometry (LC-MS), which were mapped onto the governing metabolic pathways. Notable finding in this strain concerns glycerol and CDP-DAG metabolism wherein reduced production of glycerol and phospholipids induced a bypass leading to enhanced de-novo triacylglyceride synthesis. Collectively, our findings help in understanding the central carbon metabolism of *R. toruloides* which may assist in developing rationale metabolic models and engineering efforts in this organism.

## 1. Introduction

The red yeast *Rhodosporidium toruloides* has been recognized as a promising microbial cell factory for the production of functional lipids, oleo-chemicals, and biofuels [1,2]. Triacylglycerols (TAG) is the major form of neutral lipids (up to 70% of dry cell weight) accumulated in this yeast. This basidiomycetous fungus is a potential host for metabolic engineering to produce terpenes and fatty acids [3]. Interestingly, *R. toruloides* can metabolize challenging substrates including waste glycerol, biomass hydrolysates, xylose, and can naturally co-produce valuable compounds such as carotenoids and other useful enzymes relevant to the pharmaceutical and chemical industries [2,3,4].

The fundamental requisite for yeast-based biodiesel production meeting the criteria for sustainable biofuels is the optimization of conditions that up-regulate higher lipid productivity in fast-growing oleaginous yeast. A better understanding of such phenomena leads to more accurate bioengineering of industrially viable strains. A variety of biochemical approaches have been implemented in this direction to improve biomass and lipid synthesis. Indeed, different oleaginous yeasts were investigated for high cellular lipid contents under high carbon and low nitrogen condition [5]. However, a deeper understanding of the metabolic responses to excess carbon and low nitrogen with concomitant lipid accumulation by oleaginous yeasts is still required. Nitrogen (N) limitation majorly effects central carbon metabolism and physiology because carbon and nitrogen are primary sources for the synthesis of RNA, DNA, phosphorylated proteins, and numerous ubiquitous cofactors [6].

In the past two decades, the yeast *R. toruloides* has been investigated and studied for biotechnological applications [3]. Scientific efforts concentrated not only on the production of lipid for biofuel development, but also on metabolites production, enzymes secretion and elucidating genetic mechanisms in yeast [3,7]. Nevertheless, from a microbiological point of view the metabolic flexibility and diversity of this yeast is interesting as these characteristics differentiate *R. toruloides* from many other types of yeast. However, very few reports on the potential molecular mechanisms involved in oleaginous yeast *R. toruloides* metabolic networks are available. Zhu et al. (2012) reported transcriptomic and proteomic data under nitrogen-limited conditions illustrating that lipid accumulation correlates with the induction of lipogenesis, macromolecule metabolism, autophagy, and nitrogenous compound recycling [8]. Lee et al. (2014) applied metabolomic analysis to understand *R. toruloides* carotenoid metabolism grown on glycerol during different growth phases [2]. A recent study examined the carbon metabolism of *R. toruloides* IFO0880 by using integrated transcriptomics and metabolomics approach during growth on different carbon sources (soybean oil, acetate, xylose, or glucose) [9]. In one seminal study, genome scale metabolic model of *R. toruloides* was studied to identify the gene targets for improved lipid production via metabolic engineering or by process optimization [10]. While metabolic engineering has been employed to enhance its lipid production, however, metabolism for lipid regulation still remains largely unknown. Therefore, to elucidate the biochemical mechanisms under variable culture conditions, we examined the alterations in growth, distribution of total fatty acid (FA) content and FA composition of individual lipid classes, and relative contents of metabolites relevant to culture adjustments. Despite the importance of numerous metabolic products controlling cellular dynamics and the mechanism of partitioning these metabolites into different carbon-storing molecules in yeast, their involvement in yeast physiology and in the synthesis of biofuel precursors is not well-known.

In this study, the metabolic events and physiological responses associated with the alteration in carbon and nitrogen conditions of *R. toruloides* to lipid induction were compared by metabolomics. We integrated metabolome of such data sets to delineate the molecular changes occurring within the cells upon increasing carbon percentage and N-limitation and thus gained valuable insights of the main connections en-routed to lipid agglomeration. This study enhances our understanding of the crosstalk between carbon and nitrogen metabolism with that of the microbial oleaginicity in this versatile host. Specifically, our study provided novel insights into the functional determinants of glycerol, phospholipids, TAG, γ-aminobutyric acid (GABA) and several other metabolites accumulation. Significant variations in metabolites were detected during growth on different carbon and nitrogen percentage. To understand how *R. toruloides* reprograms its metabolism to promote growth under environmental stress conditions, we mapped those modifications onto the controlling metabolic pathways. In order to improve the sensitivity and resolution of the analysis, separation techniques, such as GC-MS (gas chromatography-mass spectroscopy), HPLC (high performance liquid chromatography) and LC-MS (liquid chromatography–mass spectrometry) facilitating the identification of the metabolites during our untargeted metabolomic study were considered. The information would be valuable to create advanced cell factories by metabolic engineering for generation of lipid-derived chemical entities which are actively pursued targets due to their wide range of potential application.

## 2. Materials and Methods

### 2.1. Yeast and Culture Conditions

Oleaginous yeast *R. toruloides* NCIM-3641 was grown in yeast nitrogen base (YNB) limiting medium (Difco, without ammonium sulfate) under different condition on orbital shaker at 200 rpm, 30 °C and supplemented with glucose and ammonium sulfate accordingly. Growth was observed by UV-spectrophotometer at OD (optical density) 660 nm and dry cell weight (DCW) was determined as described previously [11]. The overnight grown cells in YPD (yeast extract, peptone, and dextrose) medium were collected by centrifugation, washed, and inoculated again in YNB medium at 0.2 OD. Initial experiments were carried out to estimate optimal glucose concentration (2% to 10% *w*/*v*) for production of biomass and lipid. After preliminary screening with carbon source, the optimal concentration of 50 g/L (5% *w*/*v*) glucose was chosen for further screening using different nitrogen sources in 100 mL YNB medium in Erlenmeyer flasks (250 mL). For this, four nitrogen treatments in YNB medium were assessed, which included 0.5% of ammonium sulfate (NH_4_)_2_SO_4_, yeast extract, urea, and peptone. All the experiments were executed for 8 days (192 h). Nitrogen treatment was selected on the basis of their high growth and lipid titer at particular interval of time for further experimental analysis. Later, (NH_4_)_2_SO_4_ showing maximum lipid titer was chosen and screened further at 1%, 0.5%, 0.12% and 0.06% concentration to achieve enhanced lipid production in *R. toruloides* 3641. Furthermore, kinetic study of *R. toruloides* was performed at three selected conditions: 3% glucose, 0.5% (NH_4_)_2_SO_4_ (control); 5% glucose, 0.5% (NH_4_)_2_SO_4_ (N-sufficient); 5% glucose, 0.12% (NH_4_)_2_SO_4_ (N-limited) for 12 days, i.e., 288 h to estimate the maximum lipid accumulating phase and glucose consumption rate.

### 2.2. Biochemical Analysis

To estimate glucose consumption, glycerol and various other extracellular metabolites, sample (1.0 mL) was centrifuged at 10,000× *g* for 5 min, syringe filtered (by 0.22 μm, Millipore Sigma, Burlington, VT, USA) supernatants were then analyzed using HPLC (Agilent, 1260 Infinity, Santa Clara, CA, USA) at a flow rate of 0.3 mL/min. For HPLC analysis, refractive index (RI) detector and Aminex HPX 87 H (300 × 7.8 mm) column (Bio-Rad, Gurugram, India) were used with mobile phase H_2_SO_4_ (4 mM) and column temperature 40 °C [12]. The sugar and other metabolites were quantified at specific retention time by dividing the sample peak area with the peak area of standard (1.0 g/L).

### 2.3. Lipid Quantification and Profiling

Modified Bligh and Dyer procedure was used for total lipid extraction and were measured gravimetrically after drying under N_2_ [13]. Briefly, 100 mg cells were collected, and glass beads (size 0.06 µm) were added with 1:2 ratio. Lipid extraction was performed by adding 2% ortho phosphoric acid (4 mL) followed by vortexing in a bead beater (5 min) for breaking down the cells. Afterwards, methanol/chloroform (2:1, *v*/*v*) were added, vortexed for 5 min and centrifuged (5000× *g*, 8 min) to separate the mixture into two phases. The upper layer was dispensed off, and thereafter, methanol/chloroform (2:1, *v*/*v*) were added again, mixed vigorously, and centrifuged at 5000× *g* for 8 min. The extracted lipids present in lower organic phase was taken out, washed with water, and dried at room temperature under N_2_ stream to evaporate the solvent completely. The lipid content (%) and lipid titer (g/L) of the oleaginous yeast were then calculated [14].

For GC–MS analysis of fatty acids, 100 mg cells were acid-hydrolyzed and transesterified with 6% sulfuric acid: methanol and heated at 80 °C for 1 h [15]. Heptadecanoic acid (50 μg) was used as internal standard. Extracted fatty acid methyl esters (FAMEs) were then analyzed by GC–MS (7890A series) equipped with Omegawax (30 m × 0.25 mm ID, 0.25 µm thickness) and 7000 GC/MS triple quadrupole system (Agilent Technologies, Santa Clara, CA, USA). NIST (National Institute of Standards and Technology) mass spectral database, AMDIS (Automated Mass Spectral Deconvolution and Identification System) and mass hunter software was used for identification and quantification of FAMEs [11].

### 2.4. Lipid Droplet Size Estimation

Lipid droplets (LD) were visualized by using confocal microscopy (Nikon, Kolkata, India) after staining the cells with Bodipy stain (0.5 µg/mL in dimethyl sulphoxide) at the excitation/emission wavelength of 480/530 nm, respectively [16]. Briefly, cells at stationary phase were harvested at 6000× *g*, 5 min and re-suspended in 50 µL of staining solution followed by 15 min incubation in dark at room temperature. Cell size and LD size were estimated by Image J software.

### 2.5. Qualitative Metabolomics Using GC-MS

For intracellular metabolites extraction of *R. toruloides* 3641, 50 mg cells at stationary phase were harvested by centrifugation at 10,000× *g* (10 min, 4 °C) and immediately quenched in liquid N_2_. Further, glass beads and 800 μL of ice-cold methanol was added for disrupting the cell membrane in a bead beater (10 min). To remove the cell debris, samples were centrifuged at 10,000× *g* for 8 min at 4 °C. The 100 µL of filtered supernatant (0.2-μm filter) was collected, vacuum-dried at 4 °C and instantly dissolved in 50 μL of freshly prepared methoxyamine hydrochloride solution (20 mg/mL in pyridine) followed by incubation at 37 °C for 90 min. Heptadecanoic acid (50 μg) was used as an internal standard. Subsequently, 99 µL of derivatizing agent N-methyl-N-(trimethylsilyl) trifluoroacetamide (MSTFA) with 1 μL of trimethylchlorosilane (TMCS) was added and incubated at 70 °C, 30 min [2]. The samples were then centrifuged (15,000× *g* for 10 min) and the supernatant was analyzed for metabolites by GC-MS/MS analysis and the data were analyzed using MetaboAnalyst 5.0 [17].

### 2.6. LC–MS Based Untargeted Metabolite Analysis

The cells at stationary phase were harvested at 4500× *g* for 15 min at 4 °C. The cell pellets were rinsed with Milli-Q water and ~500 mg of each was suspended in 500 µL of ice-cold water: methanol (20:80) solvent and stored in −80 °C overnight. After quenching, intracellular metabolites extraction of samples was performed by a bead beater for 10 min followed by centrifugation (8000× *g*, 5 min). The filtered supernatant (0.45-μm filter) was then employed for metabolites detection by Acclaim Trinity P2 (100 × 2.1 mm, 3 μm) column (Thermo Fischer Scientific, Marsiling Ind Estate, Singapore) and Orbitrap Fusion LumosTribrid Mass Spectrometer in mix mode. The LC–MS–MS data were collected (Thermo Scientific Version 3.0) using Xcalibur software. Untargeted metabolomics analysis with peak filtering compound annotation by online databases was selected as the workflow template. The buffer system and LC–MS–MS essential parameters were used as reported earlier [18].

Uncommon compounds identified under N-limiting condition of *R. toruloides* were compared to their controls in the KEGG (Kyoto Encyclopedia of Genes and Genomes) database using their KEGG IDs. These compounds’ KEGG IDs were then utilized to position them in global metabolic pathways using the Metabopathway (MetPa) analysis online tool.

### 2.7. Statistical Analysis

The standard deviation (SD) was calculated using the mean of three data obtained from all the experiments completed in biological triplicates. For significance analysis, statistical procedures such as ANOVA and *t*-test were executed using Microsoft Excel.

## 3. Results

### 3.1. Cultivation of R. toruloides at Variable Carbon Concentrations

**Figure 1:**
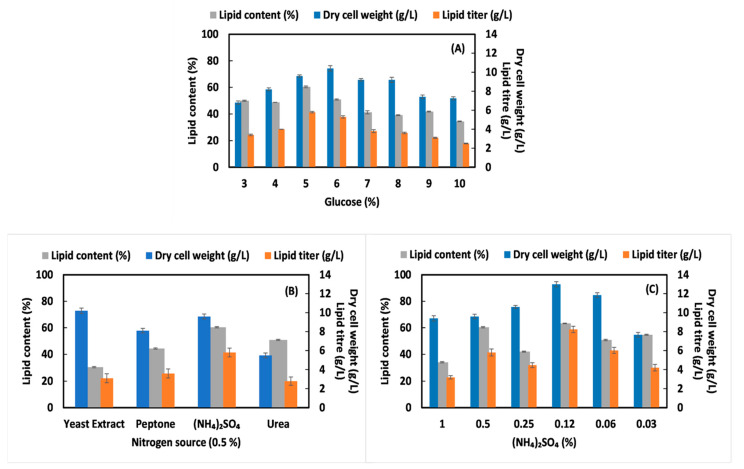
Comparative analysis of dry cell weight (g/L), lipid titer (g/L) and lipid content in *R. toruloides* at stationary phase using different media compositions (**A**) glucose (3 to 10%); (**B**) nitrogen sources (**C**) (NH_4_)_2_SO_4_ (1 to 0.03%).

Several reports concerning the lipid agglomeration of *R. toruloides* have been implemented using (NH_4_)_2_SO_4_ as the nitrogen source; however, the metabolomics study and glycerol production during the cultivation (observed in this study) has not been addressed. To evaluate whether biomass and lipid production in *R. toruloides* is influenced by the media carbon concentration, we have taken glucose concentration from 3% to 10% in YNB media and the initial nitrogen (NH_4_)_2_SO_4_ concentration was held at 0.5% (Figure 1A). When the medium was supplemented with 30 g/L of glucose, *R. toruloides* produced a DCW of 6.8 ± 0.18 g/L with a low lipid content of 50% after 144 h. At this condition, the maximum lipid was produced at 144 h (stationary phase); however, with increase in glucose percentage the carbon consumption rate decrease which led to an increase in time duration of lipid accumulating phase (Table S1).

Further, increasing the carbon concentration to 50 g/L (5%) gave a remarkable lipid content increment to 60.41% and lipid production was increased to 5.8 ± 0.08 g/L, respectively at 192 h. The cells reach stationary phase after 144 h in 50 g/L (5%) due to the presence of sufficient amount of glucose (10.52 ± 0.08 g/L) in the medium. Therefore, the cells will continue to grow at this stage and enter the stationary phase at l92 h. However, in 30 g/L (3%) carbon source only 1.6 ± 0.19 g/L glucose concentration is present at 144 h, thus limiting the cell growth which leads to early stationary phase at 144 h. Under such an excess carbon environment, *R. toruloides* showed the highest production of lipid, reflecting that carbon sources were channeled more proficiently into FA biosynthesis. Furthermore, it is worth noticing that *R. toruloides* was capable to grow when cultivated at higher concentrations of glucose (30–100 g/L) enlightening its remarkable osmotolerance. However, cellular biomass and lipid production remained low when glucose concentration increased from 6% to 10% as depicted in Figure 1A, signifying greater substrate utilization to synthesize compatible solutes balancing extra- and intracellular osmotic potential [19]. Tchakouteu et al. (2017) performed batch-bioreactor experiments of *R. toruloides* for lipid production (23.6 g/L) and found that 150 g/L of glucose with 4% NaCl resulted in satisfactory substrate acclimatization by the yeast [20].

Interestingly, with increase in the carbon percentage, glycerol production was also observed in our study which was not detected at initial % glucose concentration. Higher biomass with higher glycerol production and low lipid content was observed at 60 g/L (6%) glucose concentration in comparison to 50 g/L (5%) glucose concentration suggesting that with increase in carbon concentration, the flux is directed more towards the glycerol synthesis and less towards de-novo FA pathway as detected by HPLC (Table 1).

The glycerol produced by *R. toruloides* at 50 g/L (5%) and 60 g/L (6%) carbon concentration were 3.83 ± 0.027 g/L and 6.27 ± 0.045 g/L, respectively. In addition, the glucose consumption rate decreases by *R. toruloides* with increase in carbon percentage in the media. González-García et al. (2017) investigated that *R. toruloides* can produced maximum biomass at 100 g/L glucose and 90% consumption rate with no glycerol production [19]. Other metabolites such as citric acid and 1, 4 butanediol concentration detected were also maximum at 5% glucose and 0.5% (NH4)_2_SO_4_. This study revealed that this *R. toruloides* strain has the ability to produce lipid and glycerol simultaneously. Maximum lipid production was estimated at 50 g/L (5%) carbon; hence, this concentration is selected for further optimization of nitrogen concentration. Previous reported data showed that lower nitrogen concentration tend to improve major growth parameters and lipid yield in *Rhodotorula* sp., and enhance total fatty acid production in nitrogen-limiting cultures [8,21,22,23]. However, the mechanisms governing the positive response to nutrient downshift have never been studied which also varies among different strains. To investigate the influence of different nitrogen source and their percentage on lipid agglomeration, yeast was grown on batch cultivation conditions for 192 h.

Various inorganic and organic nitrogen sources, such as peptone, urea, yeast extract, and (NH_4_)_2_SO_4_, were employed as single nitrogen source. All nitrogen sources were shown to be capable of supporting cell growth. As demonstrated in Figure 1B, different nitrogen sources resulted in different levels of cell biomass and lipid accumulation by *R. toruloides*. The maximum biomass (DCW) was obtained in yeast extract (10.2 ± 0.28 g/L), followed by (NH_4_)_2_SO_4_ (9.6 ± 0.12 g/L) and peptone (8.1 ± 0.27 g/L), while among all nitrogen sources, the highest microbial lipid titer (5.8 ± 0.08 g/L) was achieved with inorganic source (NH_4_)_2_SO_4_. Though the higher biomass was obtained using yeast extract (0.5%) as nitrogen source, but the lipid yield was lower (3.1 ± 0.16 g/L) and the glycerol produced (6.34 ± 0.45 g/L, Table 1) was 1.65-fold higher than 0.5% (NH_4_)_2_SO_4_ representing that at this condition direction of carbon flux is more towards the glycerol pathway instead of FA. Urea resulted in a lower amount of cell biomass and lipid production. The pH of the culture broth dropped slightly at the end of the cultivation, ranging from 5.5 to 5.8, which could be due to production of organic acid during the process. Furthermore, the effect of different (NH_4_)_2_SO_4_ concentrations 1%, 0.5%, 0.25%, 0.12%, 0.06 and 0.03% at 50 g/L (5%) glucose was studied (Figure 1C). Among all, the maximum biomass (13 ± 0.52 g/L) and lipid production (8.3 ± 0.48 g/L) was obtained at 0.12% (NH_4_)_2_SO_4_ with 2.2-fold decrease in glycerol production as compared to 50 g/L (5%) glucose medium containing 0.5% (NH_4_)_2_SO_4_ (Figure 1C and Table 1). Overall, the data illustrate that 50 g/L (5%) glucose concentration and 0.12% (NH_4_)_2_SO_4_ concentrationare optimal medium conditions for higher lipid production in *R. toruloides.* We next explored time course analysis at this selected condition [50 g/L (5%) glucose and 0.12 g/L (NH_4_)_2_SO_4_] to know maximum lipid producing phase in *R. toruloides* and its comparison was undertaken with other two conditions: 50 g/L (5%) glucose, 0.5% (NH_4_)_2_SO_4_ and 30 g/L (3%) glucose, 0.5% (NH_4_)_2_SO_4_ as described briefly in the next section. Furthermore, our data indicate that metabolic engineering can be performed in this *R. toruloides* strain to divert the glycerol pathway towards de-novo lipid synthesis. To inactivate the glycerol pathway, both GPD1 and GPD2 genes encoded by the glycerol-3-phosphate dehydrogenases that are associated with the rate-limiting step of glycerol biosynthesis can be deleted [24]. Therefore, the glycerol production from carbon source can be eliminated and the carbon flux can be directed towards improved TAG biosynthesis.

In this study, both extracellular and intracellular production of glycerol has been detected in N-sufficient and N-limited conditions. However, in control (3% Glucose, 0.5% (NH_4_)_2_SO_4_) only intracellular glycerol production has been detected and not extracellular. Under excess carbon environment or external osmolarity, cells recover by accumulating compatible solutes such as glycerol for osmoregulation. The high osmolarity glycerol (HOG) pathway is activated upon hyperosmotic shock, stimulating expression of enzymes involved in extracellular and intracellular glycerol production. In the absence of hyperosmotic stress, glycerol production is required to maintain the redox balance and excess glycerol leakages out freely through the Fps1 glycerol facilitator. Intracellular glycerol produced is acylated via glycerol kinase (GUT1) to produce glycerol-3-phosphate which further leads to lipid storage pathway of TAG agglomeration. However, hyperosmotic stress causes rapid closure of the glycerol facilitator Fps1 inhibiting extracellular glycerol production [21].

### 3.2. Time Course of Lipid Accumulation under Nitrogen-Limited Condition

**Figure 2:**
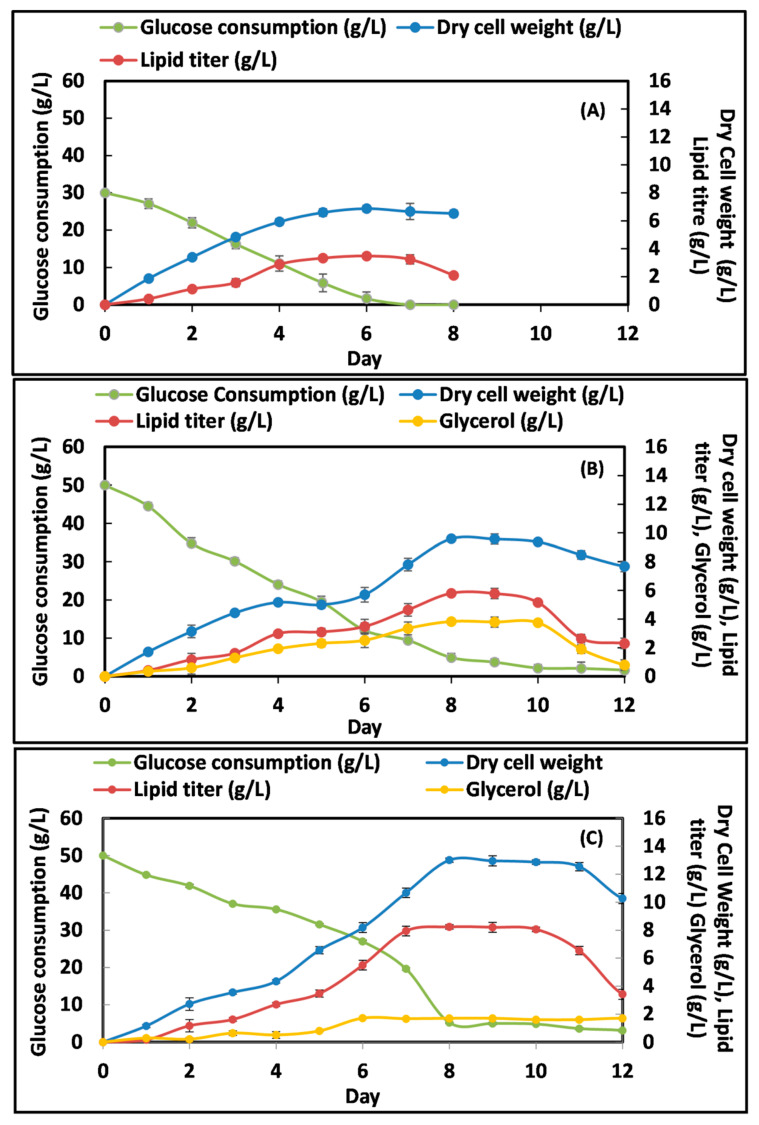
Batch cultivation experiments of *R. toruloides* 3641 for DCW (g/L), lipid titer (g/L), glucose consumption (g/L) and glycerol production (g/L) under nutritional stress conditions (**A**) control: 3% glucose, 0.5% (NH_4_)_2_SO_4_ (**B**) N-sufficient: 5% glucose, 0.5% (NH_4_)_2_SO_4_ (**C**) N-limited: 5% glucose, 0.12% (NH_4_)_2_SO_4._

To analyze cell growth, lipid production and sugar utilization patterns of *R. toruloides*, the yeast was cultivated under 0.5% and 0.12% (NH_4_)_2_SO_4_ concentrations at 50 g/L (5%) glucose. The initial 3% glucose and 0.5% (NH_4_)_2_SO_4_ condition was also used as control for comparison. Several studies have reported that biomass and lipid agglomeration in yeast depends on an adequate supply of major ions (Mg^2+^, Ca^2+^, Cl^−^, So_4_^2−^), essential macronutrient elements (carbon, phosphorus, nitrogen), as well as on micronutrient metals such as copper, molybdenum, cobalt, zinc, iron, and manganese [25,26]. To evaluate the effects of N- depletion on the growth pattern and lipid accumulation of *R. toruloides* batch cultivation experiments were performed. The results revealed that at 3% glucose concentration, the stationary phase was achieved early at 144 h but with lower lipid titer (3.3 ± 0.04 g/L) (Figure 2A). However, in N-limitation condition, maximum biomass (13 g/L) and lipid production (8.23 g/L) was estimated at 192 h (8th day) as compared to nitrogen sufficient condition (Figure 2B,C).

In addition, the glycerol production increases up to 144 h with no further increase in its synthesis. However, in N-sufficient condition glycerol production increases up to 192 h. Interestingly, no glycerol production was detected by *R. toruloides* at 3% glucose. *R. toruloides* efficiently utilized glucose for growth and lipid production as also shown in its growth curve. The time course study of nitrogen limitation medium demonstrated that biomass and lipid production were almost similar to N-sufficient condition until 96 h of growth, but then changed abruptly as the 0.12% nitrogen culture began to accumulate substantially more lipid, which finally reached 63.8% of the DCW. The lipid synthesis pathway was partially associated with the linear growth phase (15–120 h) and the early stationary phase (192 h). The sugar assimilation was 94.8% and 95.09% in N-limited and N-sufficient condition, respectively by the end of 192 h. After 120 h only 50% of the carbon source was consumed. It was investigated that carbon source was not consumed completely, which may be because essential element nitrogen had exhausted in the culture thus influencing the substrate consumption. Morphological characteristics were also found to differ during different stages of growth. The results suggested that carbon sources were mainly channeled into de-novo lipid biosynthesis pathway. Such phenomena were well in agreement with nitrogen limitation condition [8]. Interestingly, concomitant production of lipid (63.8% *w*/*w* of DCW) and glycerol has been detected in this study by *R. toruloides* 3641. In other study, an oleaginous yeast *R. toruloides* NP11 produced 33% lipid content in N-limited condition (3 mM nitrogen, 2.5% glucose) [8].

### 3.3. R. toruloideslipid Droplet Formation at Nitrogen Stress Condition

**Figure 3:**
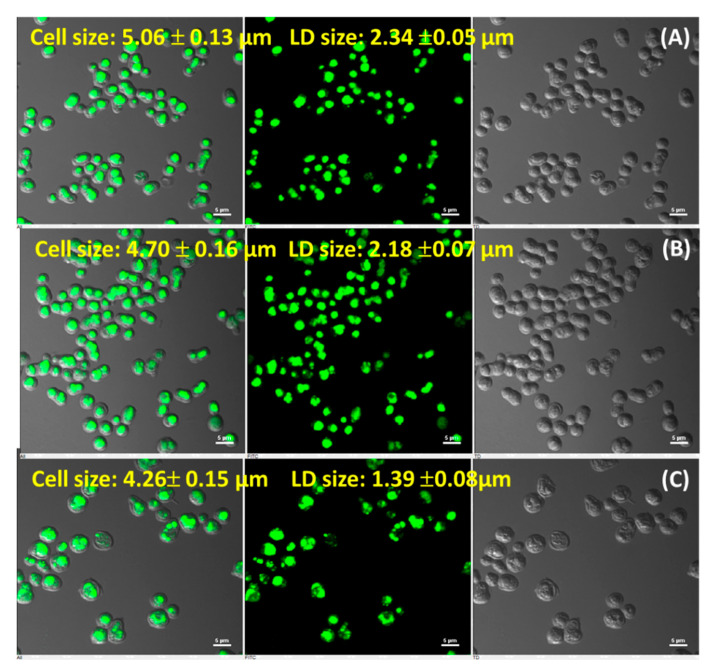
Confocal microscopy imaging for visualization of cell size and lipid droplet size in *R. toruloides* at stationary phase under different medium conditions (**A**) N-limited: 5% glucose, 0.12% (NH_4_)_2_SO_4_ (192 h), (**B**) N-sufficient: 5% glucose, 0.5% (NH_4_)_2_SO_4_ (192 h), (**C**) control: 3% glucose, 0.5% (NH_4_)_2_SO_4_ (144 h). The green fluorescence signal refers to lipid storage vesicles in the cytoplasm of *R. toruloides*.

The lipophilic (BODIPY 505/515) stained fluorescence microscopy images of *R. toruloides* at stationary phase (144 h for control; 192 h for N-limited and N-sufficient) are depicted in Figure 3. Cells grown in N-limited, N-sufficient and control were stained instinctively showing the presence of different size of LDs. The cell size and LD size of N-limited culture was larger (cell size: 5.06 ± 0.13 µm, LD size: 2.34 ± 0.05 µm) when compared to N-sufficient culture (cell size: 4.70 ± 0.16 µm, LD size: 2.18 ± 0.07 µm). In addition, the LD size evaluated in N-limited culture was 1.68-fold higher as compared to control (cell size: 4.26 ± 0.15 µm, LD size: 1.39 ± 0.08 µm). The result obtained indicates a direct correlation between LD sizes with the lipid storing ability of *R. toruloides* 3641 grown in different environmental condition.

### 3.4. Fatty Acid Profiling of R. toruloides under Nutrient Stress Condition

Fatty acid composition in yeast is known to be influenced by stress condition, particularly when deprived of certain nutrients such as phosphorus, nitrogen, or metals [25,27,28,29]. Numeral deviations take place in the overall neutral lipid content as well as the saturation profile of lipids when yeast is exposed to nutrient deprivation condition. Fatty acid methyl esters (FAMEs), i.e., biodiesel is derived during transesterification of lipids with methanol in presence of catalyst [30]. Our data depict that FAME productivities were higher in N-limited (~0.987 g/L/h) than N-sufficient (~0.696 g/L/h) at the end of the 192 h (Table 1). The FAME content per cell appears to be higher in N limited, where a constant lipid proliferation was detected, i.e., reaching up to >50% of DCW, while in N-sufficient the FAME content per cell remains low but was considerably higher than control.

**Figure 4:**
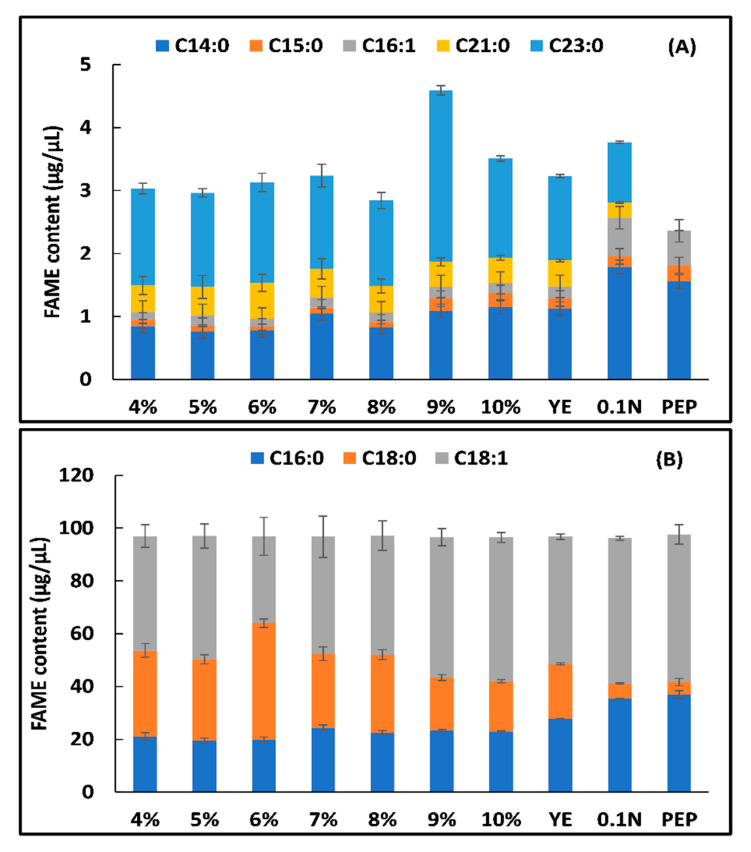
Fatty acid profile of *R. toruloides* (**A**) C14:0, C15:0, C16:1, C21:0 and C23:0 (**B**) C16:0, C18:0 and C18:1 quantified using gas chromatography-mass spectrometry (GC-MS) at different carbon and nitrogen sources (*p* < 0.05). YE represents yeast extract, 0.1 N represents 0.12% ammonium sulfate, PEP represents peptone as nitrogen source. The YNB medium with glucose 3–10% contains 0.5% ammonium sulfate and all other samples contain 50 g/L (5%) glucose.

Further, the samples subjected to nutrient stress were quantified by GC–MS analysis which revealed the presence of high amount of oleic acid (C18:1) followed by C18:0, C16:1, C14:0, C15:0, C16:0, C21:0 and C23:0 (Figure 4A,B). The amount of monounsaturated fatty acid (MUFA) on 50 g/L (5%) carbon containing 0.1% nitrogen was higher (55.65%) while amount of saturated fatty acid (SFA) was lower (44.35%) as compared to control (MUFA 52.58% and SFA 29.84%). The oleic acid content decreased with an increase in carbon concentration but further upregulated on increasing the carbon concentration from 9 to 10%. The amount of polyunsaturated fatty acid (PUFA), i.e., C18:2 was present only in control containing 3% carbon and not in other samples reflecting that increase in carbon concentration leads to down-regulation of PUFAs synthesis. When different nitrogen source was used, the MUFA content was higher on peptone (56.47%) followed by 0.1% NH_2_SO_4_ (55.65%) and yeast extract (48.35%) while SFA was higher on yeast extract (51.64%) followed by 0.1% NH_2_SO_4_ (44.35%) and peptone (43.52%). The long chain fatty acids (C21:0 and C23:0) were not detected on media containing peptone as nitrogen source as shown in Figure 4A.

The data illustrate that in N-limited condition 1.17-fold and 1.81-fold increase in oleic acid and palmitic acid content were observed, respectively as compared to N-sufficient medium. In addition, a 6.5-fold decline in stearic acid content were detected in N-limited condition in comparison to N-sufficient condition. Under N limited conditions, *R. toruloides* reveals a rise in MUFAs (55.64 % of total FAME) with considerable decrease in SFAs (44.35%) and no PUFAs content by the end of the 192 h as compared to N sufficient condition (46.85% MUFA, 53.14% SFA, 12.45% PUFA). This can be a result of the oxidative damage to PUFAs under stress or recycling of membrane lipids towards TAGs. Patel et al. (2017) reported similar increase in MUFA content (75.59%) of *R. kratochvilovae* HIMPA1 grown in N-limited condition while in P-limited condition it showed 66.79% increase in MUFA content [31]. According to the literature, *Rhodosporidium* genus produces lipids with a main proportion of unsaturated FAs having an average profile of C16:0 23–30%, C18:1 30–37%, C18:0 32–37%, C18:2 2–4% [32]. The data reveal that the FAME profile of *R. toruloides* 3641 was quite similar to that of plant oils such as cottonseed oil, soybean, and Jatropha demonstrating high degree of unsaturation which at low temperature leads to excellent fuel properties and better ignition characteristic [30,33,34].

### 3.5. GC–MS and LC-MS/MS Based Untargeted Metabolome Analysis

During the starvation condition, metabolite steps are strongly controlled to increase the survival chance of an organism [17]. Thus, qualitative metabolomics tool has been used to understand the molecular profiling under stress conditions which will offer new visions for improving the lipid production. Only a few reports on the metabolomic profile of *Rhodosporidium* sp. to understand cellular mechanism under different conditions have been studied. Here, for the first time, we employed qualitative metabolomics to explain variations in *R. toruloides* strain cultured under different glucose and nitrogen percentage, using an omics approach that offers mechanistic understanding of the carbon metabolism involved in de-novo FA synthesis. GC-MS and LC-MS/MS techniques were used and compared for their capacity to metabolite identification and sensitivity. GC-MS can detect 100–200 metabolites while more than 200 metabolites can be detected through LC-MS/MS. Moreover, GC-Ms is good for analyzing non-polar compounds as compared to LC-MS/MS. Therefore, both the techniques were used to identify maximum number of metabolites.

#### 3.5.1. GC-MS Analysis

**Figure 5:**
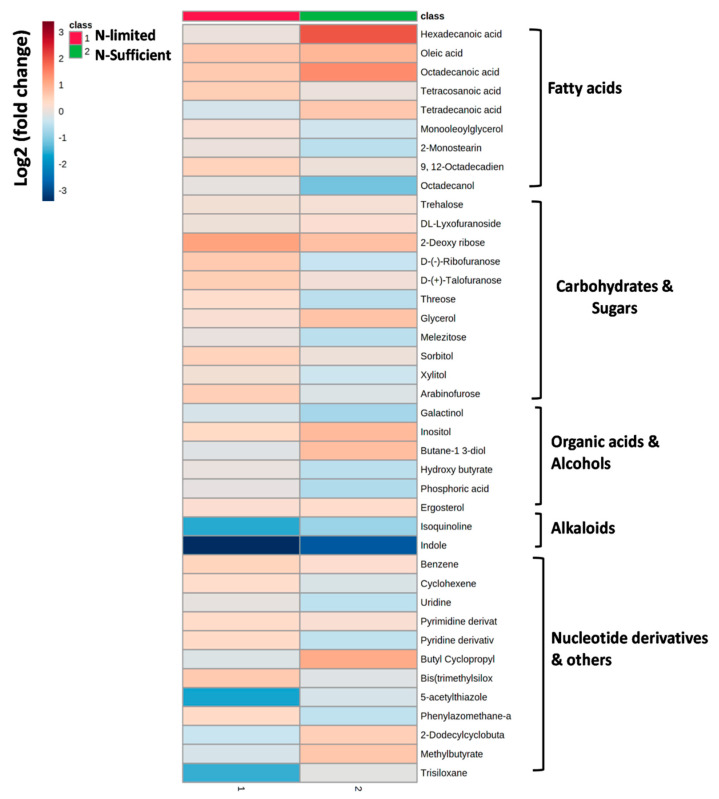
A heatmap depicting metabolites in control [3% glucose, 0.5% (NH_4_)_2_SO_4_], N-sufficient [5% glucose, 0.5% (NH_4_)_2_SO_4_] and N-limited [5% glucose, 0.12% (NH_4_)_2_SO_4_] condition at stationary phase in *R. toruloides* (*p* ≤ 0.05). The color scale represents log2 values with respect to the average value of each metabolite. Colors indicate fold increases (shown in varying degrees of red) or decreases (shown in varying degrees of blue) in concentrations, as indicated in the key.

After evaluating the variation in biomass and lipid agglomeration of *R. toruloides* under N stress, it was necessary to decipher the modifications that were occurring at the metabolic level. To categorize suppressed and induced intracellular metabolites under stress condition, we created three metabolic libraries as follows: control (3% glucose, 0.5% NH_2_SO_4_), N-sufficient (5% glucose, 0.5% NH_2_SO_4_) and N-limited (5% glucose, 0.12% NH_2_SO_4_) metabolic libraries. GC–MS data pre-processing and manual screening presented a total of 354 hits, among which 166 active metabolites were identified. A total number of 42 metabolites (Table S2) with 19 bioactive metabolites were screened and examined on the basis of their relative peak areas through KEGG database which included fatty acids, sugars, alcohols, and amino acids among which the most abundant metabolites were fatty acids. Among these 42 active compounds, 24 were identified in control, 38 in N-sufficient and 20 in N-limited. Insignificant (*p* > 0.05) and unknown compounds detected after analysis were removed. To study the relationship between GC-MS derived intracellular metabolite profiles of the samples, multivariate statistical analysis was performed by principal components analysis (PCA) score plot (Figure S1). Each dot in PCA score plot symbolize metabolites and indicates that samples in different environmental conditions were clearly separated into three groups signifying an apparent difference in their intracellular metabolism. High probabilities of their occurrence, with major number of metabolites were clustered in N-limited group. We observed about 89% of the total variance among data sets between each sample group. It displayed a high variance along the PC1 (54.1%) indicating that there was a great metabolic difference between the N-limited and the N-sufficient condition of the *R. toruloides*. The oleaginous yeast *R. toruloides* grown under different conditions was demonstrated in the form of a heat map (Figure 5) illustrating the expression pattern of various metabolites such as fatty acids, carbohydrates, alcohols sugars and organic acids.

**Figure 6:**
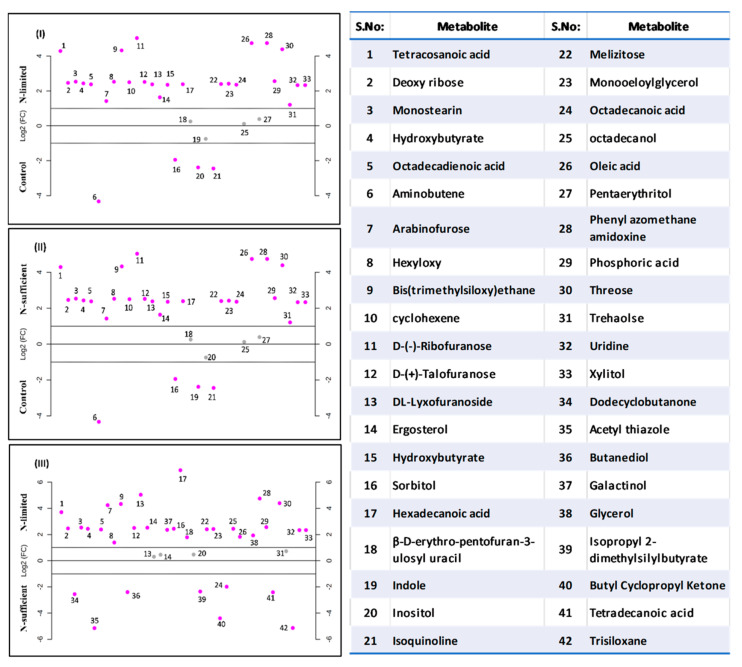
Dot-plot representing upregulated and downregulated intracellular metabolites of (**I**) N-limited with control; (**II**) N-sufficient with control; (**III**) N-limited with N-sufficient at 192 h of cultivation (grey dots denote the metabolites that show < log 2-fold change and pink dots denote the metabolites that show > log 2-fold change) in *R. toruloides*. The table represents numerical abbreviations of the metabolites. All 42 data points does not appear in all the panels as some metabolites were detected in nitrogen limiting condition which were not present in nitrogen sufficient condition or in control.

The fold change of all the intracellular metabolites in three different conditions (control, N-limited, N-sufficient) either downregulated or up-regulated are denoted in Figure 6. Many metabolites were common in all the conditions, while some of them were observed only under the applied stress condition. The fold change for all the metabolites subjected to stress conditions either down regulated or up-regulated as compared to the control. In N limited cells, many metabolites such as pyridine derivative, talose, trans-9-octadecanoic acid, linoleic acid, mannose, citric acid, valine were found to rise, while polyunsaturated fats, glucose, inositol, sorbitol were predominantly reduced. Overall, in N-limited condition intracellular metabolites such as melezitose, xylitol and ergosterol were upregulated whereas indole, inositol, glycerol and octadecanol were significantly downregulated as compared to control. Phosphoric acid intracellular metabolite lowered by 1.4-fold in N-limited cells reflecting the downregulation of cytidine diphosphate-diacylglycerol (CDP-DAG) pathway for phospholipids synthesis. In control, glycerol (3.6-fold) and trehalose (12-fold) metabolites were increased, while intermediates involved in the pentose phosphate pathway (PPP) were down-regulated. Trehalose is a non-reducing disaccharide that works as a stress signal and dehydration stabilizer, shields cellular membranes from damage, autophagy induction, involved in carbon storage and carbon metabolism and helps to preserve cell integrity [35]. It may affect glycolysis by acting as a growth regulator (via hexokinase) and leads to severe growth deficiencies. Jagtap et al. (2021) observed decrease in trehalose concentration in *R. toruloides* during growth on xylose [9]. Sugars (mannose and galactinol), which serves as a reactive oxygen species scavenger (ROS) during oxidative stress [36], is also observed to be increased in both conditions. Thus, the metabolomics profiling show that cells growing in N-limited environment suffers an abiotic stress, which leads to carbon storage in the form of sterol.

The principal FA (hexadecanoic acid, tetracosanoic acid, octadecanoic acid, octadecadienoic acid and oleic acid) gets up-regulated in N-limited condition as compared to control. However, down-regulation of butanediol and tetradecanoic acid has been observed in N-limited in comparison to N-sufficient condition. The 2, 3-butanediol production was also detected by HPLC as an extracellular metabolite, illustrating the presence of metabolic pathway involved in its synthesis from pyruvate [26]. A sugar alcohol, inositol has also been decreased by 2.40 folds in N-limitation stress condition which is an essential structural component of lipid. Improved FA production in the presence of intracellular glycerol is thought to be due to greater levels of xylulose and other transient sugars, or increased levels of pyruvate. In N-limited; stress conditions, intermediate substrate for the non-oxidative PPP such as ribose sugars were up-regulated which increases the reducing equivalents (NADPH) and glyceraldehyde-3-P (G3P). The conversion of glycerol to glycerol 3-phosphate catalyzed by glycerol kinase increases G3P pool, which further augments the lower half of the glycolysis and FA biosynthesis pathway [24]. Lopes et al. (2020) reported that *Rhodotorula toruloides* grown on acetate generates NADPH by TCA cycle dependent NADPH production pathways while glycerol grown cells use NADPH dependent glycerol dehydrogenase [10]. This significant difference in NADPH production is carbon source dependent.

#### 3.5.2. LC-MS/MS Analysis

**Figure 7:**
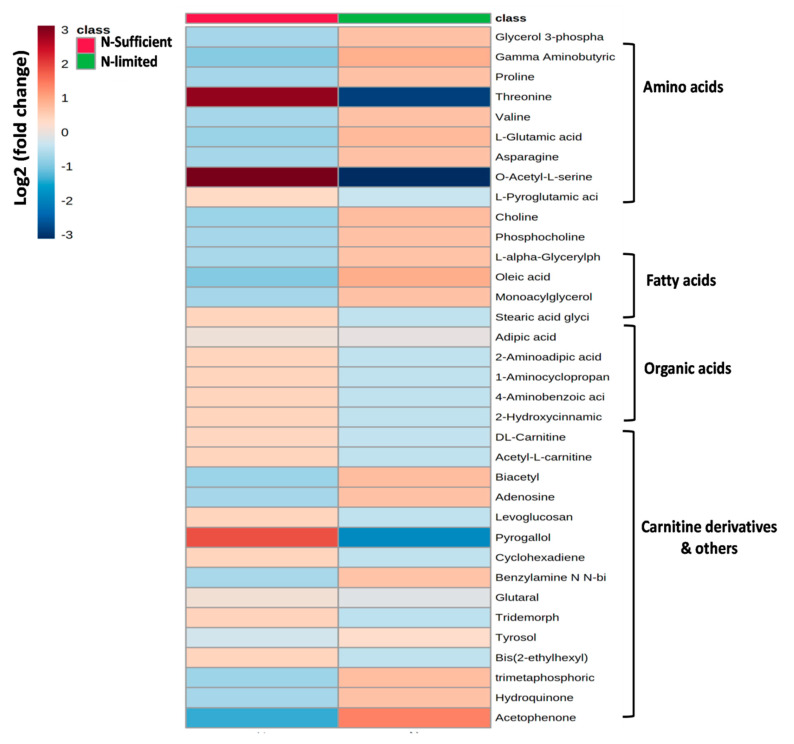
A heatmap depicting the expression of metabolites in N-limited [5% glucose, 0.12% (NH_4_)_2_SO_4_] and N-sufficient [5% glucose, 0.5% (NH_4_)_2_SO_4_] with respect to control [3% glucose, 0.5% (NH_4_)_2_SO_4_] at stationary phase in *R. toruloides* (*p* ≤ 0.05). The color scale represents fold changes with respect to the average value of each metabolite. Colors indicate fold increases (shown in varying degrees of red) or decreases (shown in varying degrees of blue) in concentrations, as indicated in the key.

Untargeted intracellular metabolites profiling of *R. toruloides* 3641 under N-limited and N-sufficient cultivation conditions by LC–MS-MS had detected 144 active metabolites. These metabolites that belong to the classes of organic acids, nucleotides, lipids, sugars, and coenzymes were subjected for multivariate analysis for comparing the metabolomic changes under N-limited and N-sufficient conditions of *R. toruloides*. Bioactive compound hits were also identified by LC–MS/MS study to support the data achieved by GC–MS. The data obtained indicates that *R. toruloides* is incapable of secreting polar molecules outside the cells. To differentiate particular intracellular metabolites detected by LC-MS that notably up-regulate and down-regulate in N-limited and N-sufficient condition, heatmap was plotted (Figure 7). These metabolites include choline and phosphocholine with less abundance in N-limited condition. The cells require choline to synthesize sphingomyelin and phosphatidylcholine which are the major phospholipids vital for cell membranes and to preserve their structural integrity. Moreover, choline also plays an essential role in lipid transport and metabolism, cell membrane signaling and modulating gene expression [31]. The metabolite of the GABA shunt pathway such as glutamate was found to be upregulated. This pathway associates with carbon and nitrogen metabolism, thereby, influencing the FA content of the yeast. Metabolites detected in this study such as glutathione, hydroxycinnamic acids, tyrosol and hydroquinone are known to exert beneficial effects linked to their antioxidant capacity protecting the cell from oxidative damage and stress. Another stress indicator, proline, maintains cells osmoregulation and NAD(P)+/NAD(P)H ratios [36]. Amino acids metabolites such as proline, asparagine, valine, and glutamic acid decreases in N-limited condition indicating the downregulation of amino acid synthesis pathway.

Furthermore, carnitine and acetyl carnitine detected were down-regulated in N-limited condition. The L-carnitine function is to transport long-chain FAs into the mitochondrial matrix for energy conversion by β-oxidation process and to remove products of metabolism from cells [9]. Moreover, acetyl-CoA and carnitine reacts together and helps in maintaining the cell acetyl-CoA/CoA ratio by regulating pyruvate dehydrogenase activity which in turn catalyzes the oxidative decarboxylation of pyruvate to form acetyl-CoA, CO_2_ and NADH (H^+^) [37]. This leads to up-regulation of de-novo FA pathway. However, in order to understand the comprehensive molecular alterations of yeast under nutritional stress, further extensive study at the proteome and transcriptome levels is required. Zhu et al. (2012) reported proteomic and transcriptomic data of *R. toruloides* NP11 and suggested that lipid accumulation under nitrogen-limited conditions correlates with the induction of nitrogenous compound recycling, lipogenesis, autophagy, and macromolecule metabolism [8]. Recently, mutant *R. toruloides* showed increased lipid production due to downregulation of mannitol biosynthesis using glycerol as carbon source [38]. In another study, LC-MS/MS-based metabolomic analysis of *Y. lipolytica* was performed for studying the impact of nitrogen-limiting conditions which revealed that pyrimidine and purine metabolism were the most highly affected biological pathways [39].

### 3.6. Metabolic Pathway Analysis

In terms of economically feasible bioenergy production, oleaginous yeast act as a favorable bio resource, but information about the cellular dynamics of yeast is not well-known. During unfavourable conditions, the excess energy gets converted into lipids and/or carbohydrates in storage form. In the present work, growth, and cellular physiology of *R. toruloides* were explained under excess carbon and nitrogen limitation condition. Perhaps the nutrient limitation stress condition can be attributed to the evolutionary behavior of oleaginous yeast; these organisms do have specific responses to conserve their structure. For the identification and investigation of metabolites from biological samples, the study was carried out to detect the variations in the metabolic pathway of *R. toruloides* grown under N-limited condition. *Yarrowia lipolytica* metabolome (KEGG) was utilized as the model organism for metabolic pathway topology and enrichment analysis as the information regarding *R. toruloides* still remain unavailable in the database.

**Figure 8:**
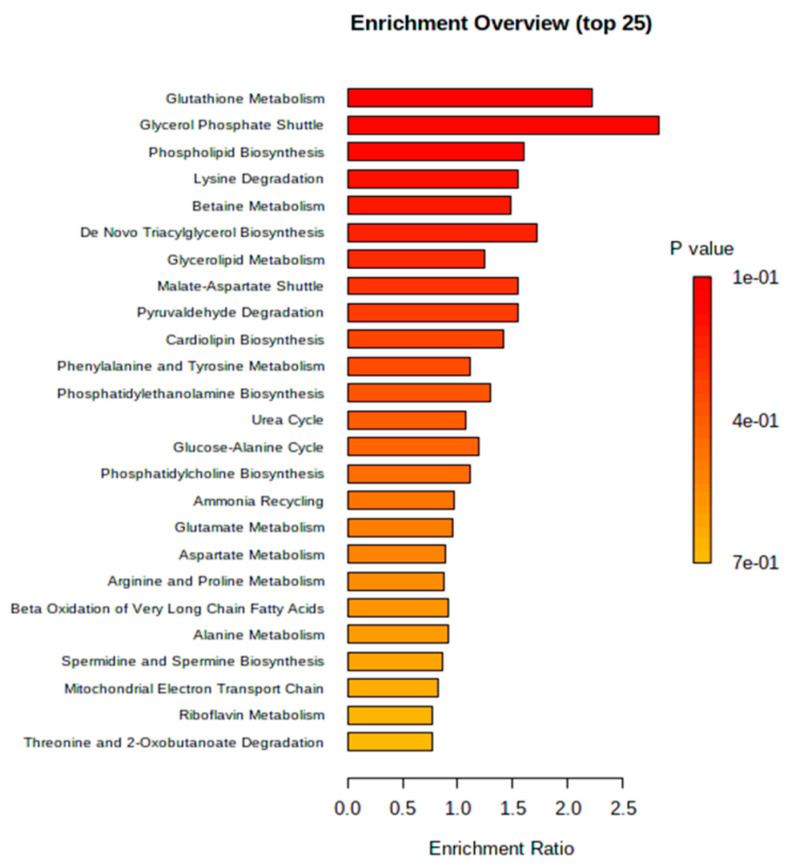
Enrichment analysis of N-limited condition [5% glucose, 0.12% (NH_4_)_2_SO_4_] metabolites classified in global pathways using *p* value and enrichment pathways. Carbon metabolism, de-novo fatty acid pathway and glutamate linked pathways showed increased *p* value with a significant increase in their fold change in the N-limited condition metabolite hits.

Combinations of metabolites found from GC-MS, LC-MS/MS and HPLC were used in the analysis. The enrichment analysis of N-limited condition metabolites classified in global pathways is depicted in Figure 8. The results show that CDP-DAG pathway of phospholipids, tricarboxylic acid cycle (TCA cycle), glyoxylate and dicarboxylate metabolism, and pyruvate metabolism were down-regulated when grown on N-limited condition (−log (*p*) > 3, pathway impact > 0.1). Subsequently, upregulation of de-novo fatty acid pathway has been achieved. Glycerol-phosphate shuttle has been highly expressed (enrichment ratio 2.5, *p* ≤ 0.05) which plays significant role in lipid synthesis. Nonetheless, other metabolism such as glycolysis or gluconeogenesis, starch, and sucrose metabolism, glycerophospholipid metabolism, amino acids metabolism (alanine, aspartate, and glutamate metabolism), β-oxidation of FA, urea cycle, electron transport chain and purine metabolism were also considerably influenced but with lesser impact.

**Figure 9:**
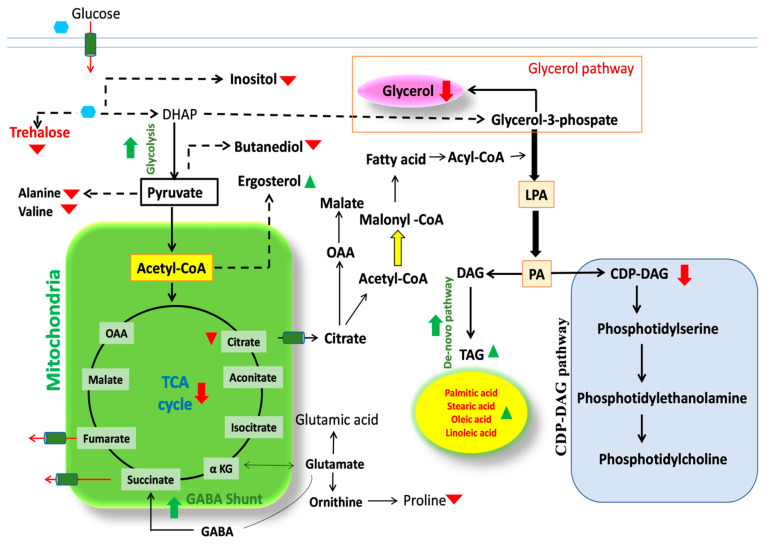
Metabolic pathway of *R. toruloides* representing diversion of CDP-DAG pathway for phospholipids synthesis and glycerol pathway into de novo TAG synthesis pathway under nitrogen limitation (red key denotes down regulation and green key denotes up-regulation).

Combination of data obtained from GC_MS, LC-MS/MS and HPLC were used for pathway construction. Metabolic pathway analysis was explored via MetaboAnalyst software based on known association with metabolites. The overall metabolic profile obtained in *R. toruloides* when subjected to nutrient deprivation has been represented in the form of a metabolic pathway (Figure 9). During N-limiting condition, cellular adenosine monophosphate (AMP) level lowers down due to activated AMP deaminase activity which catalyzes the formation of IMP (inosine monophosphate) and NH_4_ [8,37]. As a result, mitochondrial AMP level declines and ammonium level increases. This inhibits the isocitrate dehydrogenase enzyme (IDH) and blocks the TCA cycle which leads to increased citrate pool in cytoplasm. Moreover, citric acid positively controls ACCase (acetyl-CoA-carboxylase), an enzyme for the first committed step towards fatty acid synthesis. Malonyl-CoA, the first step in FA biosynthesis pathway is produced from acetyl-CoA via ACCase enzyme. Citric acid is an intermediate of TCA cycle which breaks down into acetyl-CoA and oxaloacetic acid (OAA) by ATP: citrate lyases (ACL) during transportation from mitochondria to cytoplasm, thus, diverting the flux towards TAG biosynthesis [8,40]. The non-oleaginous yeasts lack this ACL enzyme which have main role in TAG biosynthesis. In de-novo FA biosynthesis, both malonyl-CoA and acetyl-CoA reacts to form FA chain length of between 14–16 carbons. Further desaturation and elongation of FAs occurs in endoplasmic reticulum (ER). The lipid storage pathway begins from glycerol-3-phosphatedehydrogenase (GPD1) catalysed G-3-P production from acylation of glycerol via glycerolkinase (GUT1) or by dihydroxyacetone phosphate (DHAP). Phosphatidic acid produced from G-3-P is diverted into TAG and phospholipid production. ER localized CDP-DAG synthase Cds1p or mitochondrial CDP-DAG synthase Tam41p converts phosphatidic acid into CDP-DAG which is further converted into phosphatidylcholine, phosphatidylethanolamine, phosphatidylserine, and phosphatidylinositol which are the membrane phospholipids [31]. The ER-associated phosphatidic acid phosphatase (PAP) leads to the production of TAG. Under nitrogen limiting condition, phospholipids content decreases which in turn reflects that the PAP enzyme is highly active and Cds1p is less active leading to increase in TAG accumulation. It illustrates that nitrogen limitation cause’s diversion of metabolic flux from CDP-DAG pathway of phospholipids synthesis toward that TAG biosynthesis. This will cause effortless production of TAG without using inhibitors in the specific pathway or making mutants to boost de-novo TAG synthesis.

Here, one of the interesting finding concerns glycerol metabolism. Previously, *R. toruloides* was not found to produce extracellular glycerol during growth on higher glucose concentration. Glycerol is generated mainly from G-3-P via glycerol-3-phosphate dehydrogenase. TAG production also occurs from G-3-P which is found to be upregulated in this study [24]. Extracellular glycerol production is a specific characteristic of *R. toruloides* NCIM-3641 strain. Under nitrogen limitation condition glycerol production reduces in *R. toruloides* depicting that the carbon flux from G-3-P is directed towards higher TAG synthesis and less towards glycerol. Our findings suggest that metabolic engineering can be performed in this strain by inhibiting the GPD1 and GPD2 genes responsible for glycerol synthesis to further improve de-novo lipid production [24]. Taken together, these results obtained from, kinetic profile and metabolite study reveals that this basidiomycetes strain is capable of converting glucose more towards lipids production and less towards glycerol and CDP-DAG pathway under N-limited condition.

Accordingly, a crosstalk between the metabolites such as trehalose, glycerol, phospholipids, and citric acid, might be important for biomass production, glycerol synthesis as well as lipid agglomeration in red yeast *R. toruloides*. Significant metabolite changes when subjected to nutrient limitation conditions will also affect FAME productivities. As a result, our metabolomic data bestow a schematic model for understanding flux diversion that leads to alterations in lipid accumulation and FAME productivity under nutritional stress in *R. toruloides* 3641.

## 4. Conclusions

Our work shows that *R. toruloides* NCIM-3641 oleaginous yeast strain can produce 2.1-fold higher lipid titers during cultivation on 50 g/L (5%) glucose and 0.12% nitrogen as compared to control (3% glucose and 0.5% nitrogen). Boosted MUFA content of fatty acid illustrates longer oxidative stability and good low temperature performance of the fuel. Metabolomic data of *R. toruloides* demonstrated that N-limitation facilitates up-regulation of TAG biosynthesis, N-related metabolism, while down-regulation of phospholipids synthesis, glycerol synthesis, and TCA cycle thereby diverting the carbon flux towards enhanced fatty acid production. This study significantly augmented our insights on central carbon metabolism to N-limitation, microbial oleaginicity, and their crosstalk. Our results and the data sets may aid in developing genome-scale models and designing advanced cell factories of *R. toruloides* for generating various lipid derived oleo-chemicals.

## Figures and Tables

**Table 1:** Comparative study of lipid, FAME productivity and other by-products generated by *R. toruloides* during cultivation on different glucose and nitrogen concentrations.

YNB Medium	DCW (g/L)	Lipid Titer (g/L)	Glycerol (g/L)	Citric Acid (g/L)	Residual Glucose (g/L)	Butanediol (g/L)	FAME Productivity (g/L/h)
3% Glucose, 0.5% (NH4)_2_SO_4_	6.8 ± 0.18	3.4 ± 0.08	-	-	1.67 ± 0.005	-	0.53 ± 0.02
4% Glucose, 0.5% (NH4)_2_SO_4_	8.2 ± 0.15	4 ± 0.007	3.45 ± 0.038	0.52 ± 0.005	2.58 ± 0.006	0.11 ±0.005	0.48 ± 0.04
5% Glucose, 0.5% (NH4)_2_SO_4_	9.6 ± 0.12	5.8 ± 0.08	3.83 ± 0.027	0.77 ± 0.008	4.26 ± 0.007	0.12 ± 0.006	0.69 ± 0.02
6% Glucose, 0.5% (NH4)_2_SO_4_	10.4 ± 0.28	5.3 ± 0.12	6.27 ± 0.045	0.43 ± 0.007	10.29 ± 0.004	0.07 ± 0.008	0.63 ± 0.05
7% Glucose, 0.5% (NH4)_2_SO_4_	9.2 ± 0.13	3.8 ± 0.15	5.60 ± 0.036	0.49 ± 0.006	21.28 ± 0.016	-	0.45 ± 0.03
8% Glucose, 0.5% (NH4)_2_SO_4_	9.2 ± 0.27	3.6 ± 0.08	6.63 ± 0.042	0.48 ± 0.008	35.03 ± 0.015	0.07 ± 0.008	0.43 ± 0.03
9% Glucose, 0.5% (NH4)_2_SO_4_	7.41 ± 0.19	3.1 ± 0.06	3.84 ± 0.054	0.67 ± 0.009	36.89 ± 0.008	0.05 ± 0.004	0.37 ± 0.05
10% Glucose, 0.5% (NH4)_2_SO_4_	7.25 ± 0.17	2.5 ± 0.04	3.67 ± 0.044	0.70 ± 0.045	44.98 ± 0.014	0.08 ± 0.006	0.3 ± 0.04
5% Glucose, 0.5% Yeast extract	10.2 ± 0.28	3.1 ± 0.16	6.34 ± 0.45	1.10 ± 0.52	8.75 ± 0.48	-	0.37 ± 0.03
5% Glucose, 0.5% Peptone	8.1 ± 0.27	3.6 ± 0.18	2.51 ± 0.34	1.02 ± 0.37	2.60 ± 0.35	0.12 ± 0.06	0.43 ± 0.06
5% Glucose, 0.5% Urea	5.5 ± 0.14	2.8 ± 0.16	-	-	10.68 ± 0.57	-	0.33 ± 0.04
5% Glucose, 0.12% (NH4)_2_SO_4_,	13 ± 0.52	8.23 ± 0.48	1.72 ± 0.08	0.08 ± 0.004	5.2 ± 0.26	0.13 ± 0.05	0.98 ± 0.06

- Not detected.

## Supplementary Materials

The following are available online at https://www.mdpi.com/article/10.3390/jof7110967/s1, Table S1: comparative study of DCW (g/L), lipid titer (g/L) and glucose consumption (g/L) of *R. toruloides* at different time interval on various glucose concentration (0.5% (NH4)_2_SO_4_); Table S2: list of metabolites detected in *R. toruloides* under different glucose and nitrogen percentage. C: control [3% glucose, 0.5% (NH4)_2_SO_4_], 1: N-limited [5% glucose, 0.12% (NH4)_2_SO_4_], 2: N sufficient [5% glucose, 0.5% (NH4)_2_SO_4_]; Figure S1: two-dimensional principal component analysis (PCA) score plot to depict the variation among the metabolic profile of *R. toruloides* under different glucose and nitrogen percentage. C: control [3% glucose, 0.5% (NH4)_2_SO_4_], 1: N-limited [5% glucose, 0.12% (NH4)_2_SO_4_], 2: N sufficient [5% glucose, 0.5% (NH4)_2_SO_4_].

## Abbreviations

ACCase: acetyl coenzyme A carboxylase; ATP: adenosine triphosphate; ADP: adenosine diphosphate; CDP-DAG: cytidine diphosphate-diacylglycerol; FA: fatty acid; FAMEs: fatty acid methyl esters; KEGG: Kyoto Encyclopedia of Genes and Genomes; MUFAs: monounsaturated fatty acid; N: nitrogen; NADPH: nicotinamide adenine dinucleotide phosphate; PUFAs: polyunsaturated fatty acid; RPM: rotation per minute; SFAs: saturated fatty acid; TAGs: triacylglycerols; TCA: tricarboxylic acid; YNB: yeast nitrogen base.
