# Supplementary material for: Metabolomic Profiling Revealed Diversion of Cytidinediphosphate-Diacylglycerol and Glycerol Pathway towards Denovo Triacylglycerol Synthesis in Rhodosporidium toruloides
Source: J Fungi (Basel). 2021 Nov 13;7(11):967. doi: 10.3390/jof7110967 (PMC8625802; doi:10.3390/jof7110967)
Supplement: Supplementary file 1 [file jof-07-00967-s001.zip › FigureS1.pdf]

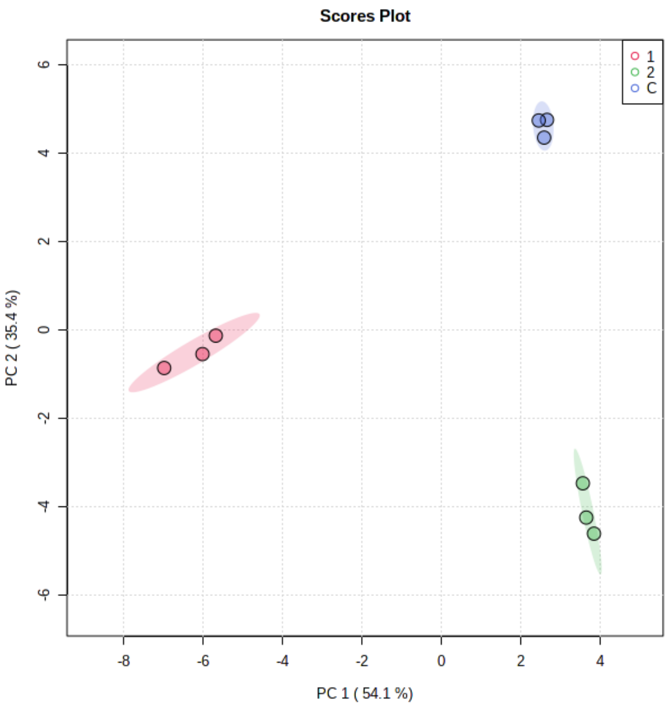

Figure S1. Two-dimensional principal component analysis (PCA) score plot to depict the variation among the metabolic profile of *R. toruloides* under different glucose and nitrogen percentage. C: control [3 % glucose, 0.5 % $(NH_4)_2SO_4$], 1: N-limited [5 % glucose, 0.12 % $(NH_4)_2SO_4$], 2: N sufficient [5 % glucose, 0.5 % $(NH_4)_2SO_4$]
