# Supplementary material for: Metabolomic Profiling Revealed Diversion of Cytidinediphosphate-Diacylglycerol and Glycerol Pathway towards Denovo Triacylglycerol Synthesis in Rhodosporidium toruloides
Source: J Fungi (Basel). 2021 Nov 13;7(11):967. doi: 10.3390/jof7110967 (PMC8625802; doi:10.3390/jof7110967)
Supplement: Supplementary file 1 [file jof-07-00967-s001.zip › TableS1.pdf]

Table S1: Comparative study of DCW (g/L), lipid titer (g/L) and glucose consumption (g/L) of *R. toruloides* at different time interval on various glucose concentration (0.5 % $(NH4)_2SO_4$)

	3 %			4 %			5 %			6 %		
**Day**	DCW (g/L)	Lipid titer (g/L)	Residual glucose (g/L)	DCW (g/L)	Lipid titer (g/L)	Residual glucose (g/L)	DCW (g/L)	Lipid titer (g/L)	Residual glucose (g/L)	DCW (g/L)	Lipid titer (g/L)	Residual glucose (g/L)
5th	6.60 ± 0.16	3.30 ±0.04	5.85 ± 0.071	5.2± 0.19	3.1± 0.20	17.34 ± 0.25	5.5± 0.06	3.1± 0.04	19.55± 0.087	6.44± 0.11	3.12± 0.14	37.08± 0.27
6th	6.80 ± 0.18	3.40 ± 0.08	1.67± 0.005	5.5± 0.32	3.48± 0.18	10.16 ± 0.35	5.7± 0.08	3.48± 0.15	12.48± 0.056	7.15± 0.28	4.27± 0.36	29.35± 0.34
7th	6.66 ± 0.12	3.23 ± 0.05	0.013 ± 0.004	7.6± 0.18	3.66± 0.16	8.23 ± 0.28	7.8± 0.05	4.64± 0.009	9.44± 0.062	9.26± 0.23	4.16± 0.34	18.61± 0.25
8th	6.53 ±0.08	2.13 ± 0.06	0	8.20± 0.15	4 ± 0.007	2.58 ± 0.006	9.6± 0.12	5.8± 0.08	4.26± 0.007	10.4± 0.28	5.3 ± 0.12	10.29 ± 0.004
9th	-	-	-	8.19± 0.18	4.02± 0.005	3.18 ± 0.23	9.50± 0.14	5.7± 0.068	3.72± 0.048	10.35± 0.045	3.3± 0.18	5.6± 0.07
10th	-	-	-	8.20± 0.26	3.99± 0.08	1.96 ± 0.24	9.38± 0.09	5.2± 0.054	2.18± 0.069	10.17± 0.34	3.2± 0.04	5.12±0.05

At 3 % Glucose, 0.5 % $(NH4)_2SO_4$ i.e. control maximum lipid is produced at 6th day (144 h) while for other conditions as the glucose concentration increases in the medium; glucose consumption rate decreases and time of lipid accumulating phase increases up to 8th day (192 h). 8th day (192 h). Hence, lipid accumulating phase is different for control (144 h) and other glucose concentration condition i.e. 192 h.

Similarly, for N- sufficient [5 % Glucose, 0.5 % (NH4)2SO4] and N- limited [5 % Glucose, 0.12 % $(NH4)_2SO_4$] maximum lipid is produced at 8th day (192 h). Hence, lipid accumulating phase is different for control (144 h) and N–sufficient (192 h) as well as N-limited (192 h) condition
